# Digital Health Technologies Applied in Patients With Early Cognitive Change: Scoping Review

**DOI:** 10.2196/82881

**Published:** 2025-12-29

**Authors:** Yunhao Zhang, Xuejiao Zhu, Shulan Yang, Arkers Kwan Ching Wong, Xinming Chen

**Affiliations:** 1 Nursing Department, Zhejiang Hospital, No. 1229 Gudun Road, Hangzhou, 310030, China, 86 18106582051; 2 School of Public Health and Nursing, Hangzhou Normal University, Hangzhou, China; 3 School of Nursing, The Hong Kong Polytechnic University, Hong Kong, China (Hong Kong); 4 School of Nursing, Fujian Health College, Fuzhou, China

**Keywords:** digital health technology, early cognitive change, Alzheimer disease, cognition, scoping review

## Abstract

**Background:**

Digital health technologies (DHTs) have the potential to revolutionize the screening, diagnostic support, monitoring, and intervention for early cognitive change. However, the full spectrum of their application and the existing evidence base in this specific patient population have not been systematically delineated.

**Objective:**

This study aimed to review and synthesize the applications, roles, and challenges of DHTs in patients with early cognitive change.

**Methods:**

This scoping review was conducted in accordance with established methodological frameworks for scoping reviews and followed the PRISMA-ScR (Preferred Reporting Items for Systematic Reviews and Meta-Analyses Extension for Scoping Reviews) and PRISMA-S (PRISMA Statement for Reporting Literature Searches in Systematic Reviews) guidelines. A systematic search was conducted across 5 electronic databases: PubMed, Embase, Web of Science, APA PsycINFO, and the Cochrane Library. The search covered the period from each database’s inception until September 30, 2025. Studies were selected, and data were extracted using the population-concept-context framework, focusing on digital health interventions for patients with early cognitive change.

**Results:**

This scoping review identified 193 studies (from 8346 initial articles, screened down to 5623 after deduplication) evaluating DHTs for early cognitive change, with a marked publication surge post 2020. Studies predominantly focused on mild cognitive impairment and subjective cognitive decline. Among the 170 studies that reported the age of participants, the mean age of the participants was 74.09 (SD 7.98) years. Furthermore, six categories of DHTs emerged: (1) artificial intelligence or big data, (2) internet of things, (3) virtual reality or augmented reality, (4) robotics, (5) mobile apps or computerized cognitive training, and (6) telemedicine. Outcomes most frequently assessed included cognitive function, mental health, and feasibility. Notably, only 23 studies measured quality of life, with limited long-term (6‐12 months) follow-up. Physiological markers, social support, sleep quality, and self-efficacy were explored but less frequently.

**Conclusions:**

DHTs demonstrate significant potential in the management of patients with early cognitive impairment, particularly playing crucial roles in screening, intervention, monitoring, and auxiliary diagnosis. This scoping review underscores that DHTs, through personalized interventions and continuous care, can effectively improve patient outcomes while innovatively incorporating the caregiver perspective. However, their practical application faces challenges in balancing technological complexity with user-friendliness. Future research needs to address five key issues: (1) the lack of long-term efficacy evidence, (2) insufficient coverage of individuals with subjective cognitive decline and caregiver populations, (3) a dearth of empirical evidence on the combined application of multiple DHTs, (4) the failure of personalized programs to fully account for individual differences, and (5) the absence of effective solutions to address data and ethical risks. There is an urgent need to establish a long-term efficacy evaluation system for DHTs through rigorous methodological validation.

## Introduction

### Background

Dementia arises from a range of diseases and injuries that primarily affect cognitive function, with Alzheimer disease (AD) representing the most common type [1]. The World Health Organization reports that over 55 million people worldwide live with dementia as of 2023, making it the seventh leading cause of death globally and a major contributor to disability and dependency among older adults [1]. The global economic cost of dementia was estimated at US $1.3 trillion in 2019 [1]. Given this substantial societal and financial burden, and in the absence of a definitive cure, effective disease management interventions are critically important [2].

According to the latest diagnostic and staging criteria for AD, the condition is categorized into 6 stages [3]. Stages 4-6 correspond to progressively worsening dementia [4]. The 0‐3 stages, including subjective cognitive decline (SCD) and mild cognitive impairment (MCI), are often regarded as the initial or transitional phases in the progression toward dementia, including AD [5]. Compared with cognitively normal older adults, individuals with SCD have twice the risk [6], and those with MCI have 4 times the risk of developing dementia [7]. These stages are critical for early intervention. However, research by Thoits et al [8] indicated that many patients are not diagnosed until the later stages of the disease, resulting in missed opportunities for early treatment and a greater burden on families [9]. Therefore, prioritizing early identification, intervention, and ongoing monitoring is essential. This scoping review collectively refers to the 0‐3 stages as the early cognitive change stages, to explore the effectiveness of early identification and interventions, and the potential of these measures in delaying disease progression and improving patients’ quality of life.

In recent years, advancements in information and communication technology have significantly enhanced the potential of digital health technologies (DHTs) in cognitive care. Compared with traditional treatments, digital health provides greater long-term benefits by incorporating smart applications, virtual and augmented reality (AR), artificial intelligence (AI), telemedicine, mobile health, chatbots, and the internet of things (IoT) [10]. These tools enable precise identification of cognitive changes and deliver personalized rehabilitation, while real-time data allows health care professionals to optimize interventions and improve patient outcomes [11,12].

Current methods for identifying and intervening in cognitive decline are diverse, with numerous DHTs emerging as alternatives or supplements to traditional cognitive therapy. These technologies have been widely studied; however, the research and development efforts are often fragmented. Existing reviews tend to focus on specific DHTs used in patients with MCI or AD, such as AI [13], virtual reality (VR), and mobile apps [14]. While these studies contribute to understanding the application of individual tools in managing early cognitive change and dementia and promote technological optimization, they fail to provide a comprehensive overview of the broader application landscape of DHTs across the spectrum of early cognitive change.

### Research Problem and Aim

A comprehensive understanding of DHTs in early cognitive change remains incompletely developed, largely due to a fragmented and narrow evidence base. Previous reviews have predominantly addressed limited efficacy questions, failing to provide a consolidated overview of the broader application landscape. To address this gap, this scoping review was designed to systematically map the entire field. This scoping review aims not only to catalog the diverse types of DHTs, the application scenarios, and target populations but also to synthesize key challenges identified across the literature. By doing so, it seeks to establish a foundational understanding for future research and to inform the integration of DHTs into clinical and health care systems. This scoping review provides a systematic synthesis of the evidence on DHT applications in early cognitive change and a clear delineation of prevailing challenges.

## Methods

### Overview

This scoping review followed the framework outlined by Arksey and O’Malley [15] with an extended version by Levac et al [16], and was reported according to the PRISMA-ScR (Preferred Reporting Items for Systematic Reviews and Meta-Analyses Extension for Scoping Reviews) [17] and PRISMA-S (PRISMA Statement for Reporting Literature Searches in Systematic Reviews) [18,19], with the completed PRISMA-ScR checklist (Checklist 1) and PRISMA-S checklist (Checklist 2).

### Identification of the Relevant Studies

A comprehensive 3-stage search strategy was implemented. First, preliminary searches in PubMed and Embase databases were conducted to explore the application of DHTs in patients with early cognitive change. This scoping review then analyzed the text words in the titles and abstracts of the retrieved records, along with their relevant index terms. Second, based on this analysis of frequently used terms and concepts, a systematic search was conducted across 5 electronic databases: PubMed, Embase, Web of Science, APA PsycINFO, and the Cochrane Library. Each database was searched individually from its inception until the final search date of September 30, 2025; all searches were last updated and rerun on this date to ensure timeliness. The complete search strategies are provided in Multimedia Appendix 1. Third, the reference lists of all identified studies were screened, and a supplementary search was conducted via Google Scholar to locate additional relevant publications. For studies whose full text was unavailable, this scoping review attempted to retrieve them by contacting the corresponding authors, relevant experts, manufacturers, or through other available channels. The search strategy was developed independently for this review and was not adapted from any previously published work.

### Study Selection

The inclusion and exclusion criteria were developed based on the population-concept-context framework.

1. Population: According to the latest AD diagnostic criteria [3], this scoping review included patients in stages 0 to 3, including those with SCD, MCI, or preclinical AD, as well as their caregivers, including family members or spouses.
2. Concept (intervention): This scoping review focused on studies involving various types of DHTs, including IoT, AI, VR, AR, robotics, data science, big data, smartphone apps, computer applications, and telemedicine. Studies that did not involve interaction with DHTs, such as computer applications that merely involve watching stimuli, were excluded.
3. Concept (outcome): Outcome measures related to the intervention were included, whether assessed through self-reporting or objective assessments conducted by clinicians, researchers, or caregivers. These measures included cognitive function, physical function, mental health, sleep quality, quality of life, physiological markers, sociability, task performance, feasibility, model evaluation metrics or diagnostic accuracy, caregivers’ burden, and others. Studies that focused solely on the architecture or implementation of devices, without assessing the impact of the intervention on users, were excluded.
4. Context: Only articles published in English were included. Eligible study types included randomized controlled trials (RCTs), quasi-experimental studies, cohort studies, case-control studies, cross-sectional studies, case reports, and descriptive studies. Studies such as reports on DHTs without patient outcomes, dissertations, study protocols, trial registrations, conference abstracts, editorials, and letters were excluded.

Studies retrieved from the databases were filtered according to the eligibility criteria outlined above. The requirements were based on the population-concept-context framework, as this scoping review aimed to examine the scope and outcomes of various DHTs. Only studies in which the keywords corresponded to the population, intervention, and outcome were included, while studies that mentioned these keywords only in the background of their research were excluded.

Search results from each database were merged using EndNote X9 (Clarivate). After duplicates were removed, 3 independent researchers (YZ, SY, and XC) screened the studies based on titles and abstracts and reviewed the full texts of the remaining studies according to the eligibility criteria. Any disagreement between the researchers was resolved through discussion.

### Data Extraction

A data extraction form was developed based on the Joanna Briggs Institute methodology guidelines for scoping review [20]. The form was piloted using 5 randomly selected articles and refined before full implementation. The extracted data included authors, year of publication, study design (eg, RCT, quasi-experimental studies, cohort studies, case-control studies, cross-sectional studies, case reports, descriptive studies, and qualitative research), participant characteristics (including demographic features and stages of cognitive impairment), type of DHTs, and outcome measures. Furthermore, 2 independent reviewers (YZ and XC) conducted the data extraction, with a senior reviewer (SY) consulted to resolve any discrepancies.

### Collating, Summarizing, and Reporting the Findings

Based on the extracted data, a descriptive analysis was performed to examine trends and the distribution of DHTs across different application purposes, such as screening, diagnostic aid, intervention, and monitoring. The outcomes related to DHTs used in early cognitive change were evaluated, and studies were categorized by their application purposes. The findings were then summarized and presented descriptively through tables and figures to address the review questions.

## Results

### Literature Search

**Figure 1.**
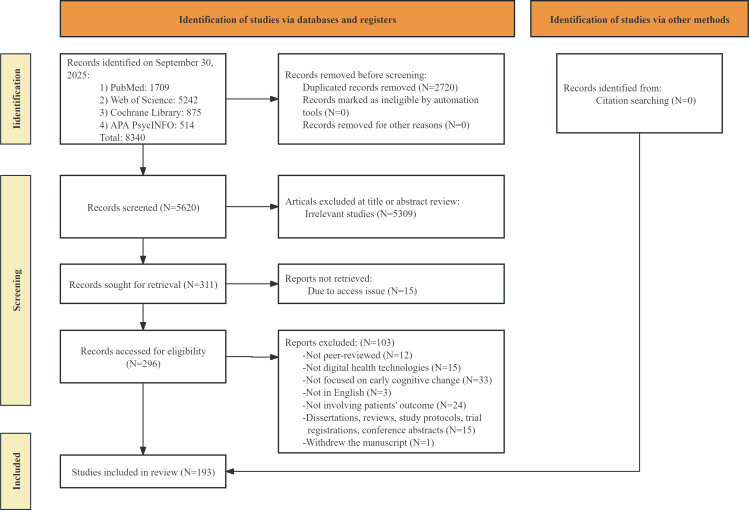
PRISMA (Preferred Reporting Items for Systematic Reviews and Meta-Analyses) flow diagram for study selection.

The detailed study selection process is presented in Figure 1. A total of 8340 articles were identified, and 5620 were retained after excluding duplicates. According to the eligibility criteria, 5309 articles were removed during title and abstract screening, 15 articles could not be accessed, and 100 were excluded after full-text review by 2 researchers (YZ and XC). The final selection for this scoping review included 193 articles [21-213] (Figure 1, Checklist 1).

### Characteristics of the Included Studies

**Figure 2.**
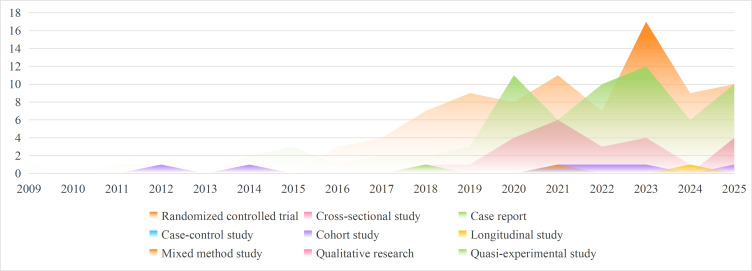
Trend analysis of different study designs in DHTs applied in patients with early cognitive change (2009‐2025). DHT: digital health technology.

The publication trend (Figure 2) of DHTs applied to early cognitive change showed a notable increase from 2020 onwards. Although the first relevant RCT [108] was published in 2009, the number of studies on DHTs remained relatively stable until 2020. Subsequently, there was a marked surge in publication volume, with 26 studies [33,39,43,48,51,57,61,62,68,75,76,78,82,86,92,98,102,134,136,149,155,162,167,168,170,181] published in 2021, 21 studies [21,25,32,38,42,60,65,72,93,95,100,113,117,119,120,132,141,144,176,180,183] in 2022, 33 studies [24,26,27,35,36,41,44,46,47,56,59,66,69,80,81,88,99,106,114,128-131,138,142,153,157,161,163,173,185,190,202] in 2023, 17 studies [23,97,101,110,121,123,140,143,145,146,154,166,172,197,200,201,203] in 2024, and 23 studies [79,124,186-189,191,193-196,198,199,204-213] in 2025. Experimental studies constituted the majority (157/193) of the research identified as of September 2025. Among these, case report, case-control study, longitudinal study, and mixed method study each constituted only 1 included study [76].

The studies predominantly focused on patients with early cognitive change, particularly those with MCI and SCD. The majority of the studies focused on patients with MCI (110/193). Only 10 studies [30,33,46,55,68,75,82,133,138,167] examined patients with SCD, with 4 studies [30,75,82,138] focusing solely on this group. Only 6 studies [65,91,92,154,193,196] included informal caregivers of the patients. Regarding the age criteria for inclusion, the age range across all studies spanned from 50 to 96 years. Some studies did not use age as an inclusion or exclusion criterion; instead, they used cognitive function scores (such as Mini-Mental State Examination and Montreal Cognitive Assessment) for participant screening. Based on the available data from studies that reported mean age, the overall mean age of participants across the included studies was calculated to be 74.09 (SD 7.98) years.

### Type of DHTs Implemented in Patients With Early Cognitive Change

**Figure 3.**
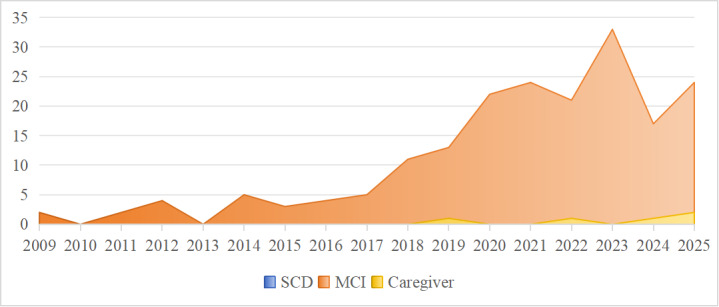
Distribution of study populations in DHT research: patients with MCI, patients with SCD, and caregivers (2009‐2025). DHT: digital health technology; MCI: mild cognitive impairment; SCD: subjective cognitive decline.

**Figure 4.**
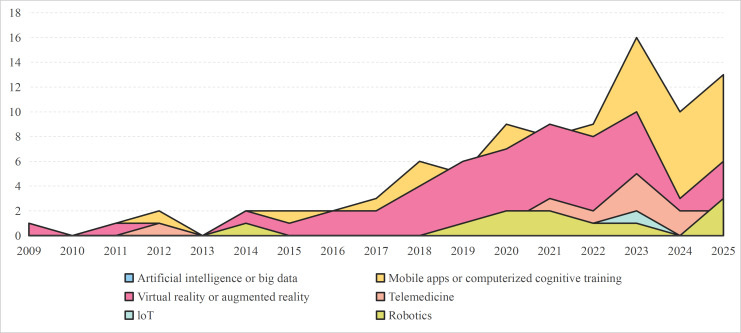
The evolving landscape of DHTs in early cognitive change research: trends in the use of 6 types (2009‐2025). DHT: digital health technology; IoT: interet of things.

DHTs are increasingly being used in patients with early cognitive change. Given that AI and big data technologies are data-driven, while smartphone apps and computer applications serve as application-based tools, this scoping review categorized these technologies into 6 main types: AI or big data, IoT, VR or AR, robotics, mobile apps or computerized cognitive training, and telemedicine. The distribution of research subjects related to DHTs is illustrated in Figure 3, while the development trends of various DHTs are depicted in Figure 4.

Research primarily focused on mobile apps or computerized cognitive training, with 89 studies [24,25,28,30,32,40-42,44,46,57-59,65,67,69,77,78,80-82,84,85,94,96,98,99,101,103-105,107-117,120-122,124-126,128-131,133,138,140,141,145,147-152,154,156,159,162,164,170-172,180,181,188,189,193,196,197,199,201-206,208-211] examining its impact. The majority of these studies used computer programs or mobile apps (78/89), while a smaller number used gaming consoles (8/89) or web-based platforms (3/89). It is noteworthy that in specialized studies targeting individuals with SCD, half of them used mobile apps or computerized cognitive training interventions. These data indicate that smartphone apps and computer applications not only possess broad applicability in the field of cognitive intervention but, due to their user-friendliness, have also become the preferred technological medium for populations experiencing early cognitive changes such as SCD.

VR research also expanded, with 63 studies [21,26,27,33,36,37,43,50-56,60,68,71,74-76,83,86-89,100,106,118,119,123,127,132,134,135,137,142,144,153,155,158,160,165,166,169,173-179,182-185,187,192,194,195,200,207,212,213], particularly since 2017, VR technology has been applied in a variety of scenarios, including virtual cycling, road crossing simulations, and virtual supermarkets, all aimed at enhancing cognitive training through immersive environments. Among these apps, the virtual supermarket is the most commonly studied scenario, with 19 studies [33,36,43,50-52,54-56,68,75,86,89,119,127,142,144,175,176] dedicated to its construction or validation. Some studies, such as the one by Afifi et al [21], integrated multiple VR activities, such as virtual adventures, recalling life stories, and virtual photo videos, to target various cognitive domains to improve overall cognitive function.

A total of 14 studies [25,45-49,61-63,81,102,139,157,198] on AI or big data primarily implemented machine learning techniques (eg, support vector machines, random forests, and deep learning) to detect early cognitive change and personalize interventions. Furthermore, 2 studies [48,180] also applied natural language processing to enhance detection and intervention precision.

In robotics, 10 studies [22,34,70,72,73,161,167,168,186,191] explored social assistance and gait training; 7 studies [34,70,72,73,167,168,186] focused on humanoid robots designed to improve social skills and cognitive function, while 1 study [22] developed omnidirectional mobility robots for physical rehabilitation [22].

IoT research is limited, with 11 studies [23,26,31,66,90,91,95,97,153,198,209] on patient monitoring via devices, such as wearable cameras, wrist devices, and smart home sensors. These technologies enable continuous, comprehensive monitoring of patients’ cognitive and physical states [23].

Telemedicine saw a rise in research, with 14 studies [24,29,35,38,39,64,79,92,93,136,143,146,163,190] conducted, particularly after 2020. Some studies have integrated various DHTs to enhance telemedicine interventions. For example, the study by Nousia et al [24] combined an app-based cognitive training (smartphone apps or computer applications) system with telerehabilitation, while Fristed et al [25] explored the use of smartphones with deep learning algorithms to improve recall tasks. Additionally, Tian et al [26] incorporated IoT devices and VR interventions to create a more immersive and effective therapeutic approach.

### Outcomes of DHTs in Patients With Early Cognitive Change

**Figure 5.**
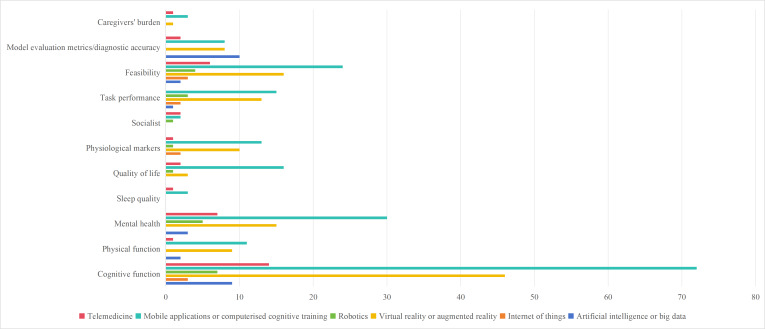
Mapping reported outcomes to DHT types in early cognitive change research (2009‐2025).

Figure 5 highlights the application of DHTs in managing early cognitive change, illustrating their impact across various outcome measures. Notably, most studies used multiple outcome measures, with cognitive function (150 studies [24,25,27,29-31,33,35,36,38,40-44,46,48,52,55,59,61-63,65-70,72,73,75,78-87,89-119,121-123,125-140,142-153,155,156,158-160,162-167,169-171,173,175,177-179,181,183-192,195,197,199-205,207-213]), mental health (62 studies [21,23,27,28,30,31,33,34,36,38,40,42,43,46,47,55,59,67,68,70,73,77,81,85,90-94,97,104,105,108,109,111,116,117,123,127,131,134,136,138,142,144,146,149,152,164,167,185,189-192,195,197,203,205,206,208,211]), and feasibility (53 studies [21,22,25-27,32-35,37-40,42,66,67,76,85,88,91-93,99,107,110,111,115-117,127,134,138,140,142-144,152,159,168-170,172-174,176,180,181,184,186,193,196,198,209]) being the most frequently evaluated.

The included studies used a diverse array of cognitive assessment tools to measure various cognitive domains in patients with early cognitive changes, encompassing overall cognitive function, memory, executive function, attention, processing speed, and language abilities. The selection of assessment tools for cognition varied across different studies. Traditional assessment tools, such as the Mini-Mental State Examination, Montreal Cognitive Assessment, or Trail Making Test, were the most commonly used. Additionally, some studies collected data on gait, speech speed, and facial expressions and developed novel digital cognitive assessment tools. Compared with traditional tools, these digital technology-based tools minimize examiner bias and practice effects through standardized administration and adaptive item pools, enabling long-term, frequent home-based monitoring. Notably, a small subset of studies determined patients’ cognitive function based on observational descriptions provided by family members.

Negative emotions, such as depression, anxiety, and apathy, are often linked to reduced cognitive function [27]. The research by Afifi et al [21] explored how VR technology enabled adult offspring to remotely participate in parental care by recreating familiar locations for shared activities via VR. The study found that VR not only enhanced the psychological well-being and relational dynamics between older adults and their family members but also improved the quality of life for older adult patients while reducing caregivers’ guilt. Chandler et al [28] evaluated 5 interventions (yoga, computerized cognitive training, health education, support groups, and memory support system) and demonstrated that persistent computerized cognitive training for 12 months significantly reduced anxiety levels while enhancing self-confidence. Furthermore, wearable devices were used to track neuropsychiatric symptoms, with findings suggesting a relationship between anxiety, sleep quality, and cognitive outcomes [23].

Sleep quality was often monitored using IoT and wearable devices in many studies, revealing a connection between sleep and cognitive improvement [29,30]. Notably, studies examining sleep-related issues consistently assessed mental health alongside sleep metrics [23,31].

Feasibility outcomes—assessing factors such as usability, adherence, and patient satisfaction—were generally positive, with high participation rates, especially for interactive interventions [32]. However, challenges like mild VR-induced dizziness [33], low engagement in robot-assisted training [34], and poor telemedicine connectivity [35] were noted but were expected to diminish over time [36]. Overall, VR-induced vestibular symptoms, connectivity issues, and digital literacy gaps among the older adult population were reported when discussing the negative outcomes when applying DHTs.

Physical function was assessed using indicators including balance, gait, walking speed, muscle strength, flexibility, activities of daily living (ADLs), and fall risk. Studies showed that gait changes are often used to screen for early cognitive change, and interventions using VR or telemedicine could significantly improve balance [37], gait [38], and reduce fall risk [39]. ADLs, key in assessing physical function, are divided into basic [40,41] and instrumental [42,43] tasks, with most studies focusing on the more complex instrumental ADLs. The results indicate that the ability to perform ADLs can be improved through intervention.

Several studies developed screening tools using DHTs to address the limitations of current methods. These studies often used model evaluation metrics such as sensitivity, specificity, accuracy, area under the curve, *F*_1_-score, and precision as outcome measures. Data sources included voice [25,44-48] and video recordings [49], facial expressions [47], behaviors in VR [43,50-56], app usage [57-61], gait tests [62,63] (stride time, step length, speed, and support times), and general scales. Most studies used machine learning for data analysis, while others used simpler statistical approaches, like logistic regression [43,53,59-61,64,65].

Task performance was commonly used as an outcome measure in VR and smartphone app and computer application interventions, and it was typically assessed through metrics such as completion time, accuracy, and error rate in memory tasks. This outcome measure was highly flexible, offering immediate feedback that helped patients self-adjust and allowed researchers to observe the direct effects of the intervention.

Cognitive interventions typically aim to improve quality of life by enhancing cognitive abilities. Twenty-three studies [21,28,29,37,38,40,42,65-67,94,99,104,105,111,115-117,134,138,191,197,211] used quality of life as an outcome indicator. While most studies indicated that cognitive improvements are associated with better quality of life, this relationship is not always significant [66]. It is important to note that only 3 studies [28,65,67] investigated the quality of life at 6 or 12 months postintervention, with the majority focusing on shorter follow-up periods (3‐4 months), leaving the long-term effects largely unexplored.

Other outcome measures include physiological markers, social connectedness, self-efficacy, and personal habits. Physiological markers are typically assessed using wearable devices, such as an electroencephalogram [68] and magnetic resonance imaging [69]. Although less explored, DHTs showed promise in reducing social isolation and improving social connectedness [70]. Self-efficacy [214] and personal habits [71] were also investigated as indicators of intervention success.

## Discussion

### Principal Findings

Based on the scoping review of the application trends of DHTs in populations with early cognitive changes, this scoping review found that the number of relevant studies increased significantly since 2020. The technical types covered 6 major categories, including AI or big data and VR or AR. However, this scoping review revealed an imbalance in the target population: patients with MCI were the main focus, while insufficient attention was paid to individuals with SCD and caregivers. The outcome measures focused on cognitive function, mental health, and feasibility. Building on the study results, this scoping review discussed and sorted out the application scenarios, aiming to provide a comprehensive perspective for health care professionals, policymakers, and technology developers to guide future research and innovation in this field.

### The Application of Various DHTs in Early Cognitive Change

The discussion section of this scoping review focuses on the current application status and challenges of DHTs in patients with early cognitive changes, revealing the technology characteristics and clinical needs through systematic comparison.

Although robotic technology can enhance motor rehabilitation and assist in diagnosis through physical interaction [22,72,73,215], its high cost and the risk of replacing human interaction [34] stand in stark contrast to the multisensory immersion advantages of VR technology. VR technology can more naturally activate the episodic memory of patients with early cognitive changes by reconstructing virtual environments (such as recreating home scenarios, virtual grocery shopping in extended time, etc) [74]. When combined with personalized intervention strategies, it can enhance self-efficacy [75]. However, it should be noted that the motion sickness induced by VR may offset some of its cognitive benefits [76]. This complementary nature of “blending the virtual and the real” suggests that future research could explore the potential of hybrid systems combining robotics and VR.

Mobile apps or computerized cognitive training interventions encompass software that runs on computers, mobile phones, tablets, or gaming consoles. These tools are user-friendly and easily accessible. However, research findings indicate that their effectiveness hinges on patients’ levels of engagement and technological familiarity [77]. Taking Wii Sports (Nintendo EAD) games as an example, their cognitive training efficacy not only depends on the difficulty gradient of the game design but is also significantly correlated with patients’ digital literacy levels [32,78]. It is recommended that, in the future, before implementing interventions using smartphone apps or computer applications, a comprehensive assessment should be conducted on patients. This assessment should screen patients’ baseline cognitive abilities, evaluate their operational proficiency with digital technologies, and track their continued willingness to participate after the intervention, thereby forming a closed-loop intervention pathway.

Telemedicine demonstrates cost-effectiveness advantages in remote areas [29,38]. However, its effects have a double-edged nature. While it can overcome geographical barriers, it may potentially exacerbate anxiety and depression in certain scenarios [29,79,80]. The IoT technology enables continuous health monitoring through multidevice collaboration, coupled with rapid analysis of digital biomarkers by AI, showing immense potential in the detection of early cognitive changes [25,47,62]. Nevertheless, the reliance of AI on large datasets and challenges in interpretability restrict its application effectiveness in resource-constrained environments [81].

Future research should focus on technology integration and the practice of the 3C principles: compatibility (evaluating the match between technological interfaces and patients’ physiological characteristics), continuity (dynamically adjusting intervention programs in accordance with disease progression), and cost-effectiveness (quantifying the ratio of technological investment to clinical benefits). This integrated deployment strategy not only addresses the need for improvement regarding the current inadequacy in cross-comparative studies of DHTs but also establishes a complete logical chain from current situation analysis to decision-making frameworks.

### The Potential Application of Specific DHTs to Aid Those With Cognitive Complaints

#### Screening

The goal of screening is to detect health issues early, facilitating timely intervention and halting disease progression [216]. IoT and AI leverage wearable devices and smart home sensors for continuous monitoring of cognitive and behavioral patterns, such as gait, sleep, and activity levels [31]. AI algorithms can identify early cognitive change [25]. This passive monitoring enhances early screening capabilities. Concurrently, telemedicine apps and VR-based tests, like the virtual supermarket test, actively assess cognitive function through remote interactions and simulated environments [56,82]. These combined passive and active approaches offer a comprehensive strategy for the early detection of cognitive decline.

#### Diagnostic Support

This scoping review explored the role of DHTs in assisted diagnosis, which enhances diagnostic efficiency and supports clinical decision-making without replacing physicians’ judgment. VR techniques, such as the Virtual Supermarket Test [68], Virtual Goal Orientation [74], and Virtual Fire Drill [83], are instrumental in assessing cognitive function, mental health, and physiological markers, aiding in the distinction between patients with SCD, MCI, and AD. Social robots were used to facilitate the diagnosis of cognitive changes in patients with MCI [72], while apps were developed to automatically analyze speech and language patterns in individuals with MCI [46]. Furthermore, an internet-based telerehabilitation system was used for diagnosing swallowing disorders in patients with MCI [64]. These digital diagnostic aids excel in processing complex data and presenting comprehensive results to health care professionals, thereby improving diagnostic accuracy and reducing physician workload.

#### Intervention

The application of DHTs in interventions for early cognitive change aims to slow or improve cognitive decline, enhancing patients’ quality of life and functional independence.

Among these technologies, smartphone apps and computer applications are widely used, offering individualized programs that target various cognitive domains such as memory, attention, and executive function. These programs, which include somatic games [84], computer applications [40], and story recall exercises [85], are designed to meet the specific needs of each patient.

VR is another promising tool for cognitive training, providing immersive environments that simulate real-world scenarios, such as virtual supermarket shopping [86], driving [71], and road crossing [87]. VR offers multisensory stimulation, enhancing memory, attention, spatial navigation, and executive functions by integrating visual, auditory, and tactile feedback [88]. Unlike traditional methods, VR is more interactive and adaptive, allowing task adjustments and providing immediate feedback, which significantly boosts training effectiveness [88,89].

Telemedicine, which facilitates remote diagnosis and rehabilitation, enables patients to access health care services from home while maintaining interaction with health care providers. By leveraging videoconferencing, remote monitoring, and online consultations, telemedicine allows for real-time assessment, treatment plan adjustments, and cognitive training via digital platforms like smartphone apps and computer applications. This approach offers essential support, especially for patients with mobility impairments [24].

Robotics also plays a key role in slowing cognitive decline. Social robots, which assist with cognitive training and provide emotional support, are particularly valuable. These robots engage in verbal interactions, supporting language training, offering daily reminders, and assisting with tasks like medication management and exercise routines. Such functionalities increase the acceptability of robots among patients and their families [22,34].

In summary, the integration of various DHTs offers significant promise in addressing early cognitive change, enhancing training effectiveness, and improving the overall care experience for patients.

#### Monitoring

Monitoring aims to continuously track changes in the individual’s health and cognitive function, allowing for the early identification and management of potential issues. Long-term data collection and analysis provide insights into the progression of cognitive impairment and treatment effectiveness, enabling the development of more personalized and adaptive care plans. Only 4 studies [23,90,91,95] on monitoring were identified, all focused on IoT and wearable devices. For instance, environmental sensors continuously track conditions like light, noise, temperature, humidity, carbon dioxide, and air quality to identify factors affecting cognitive function and behavior [23]. Wearable sensors can monitor physiological signals, such as heart rate, heart rate variability, and electrodermal activity, during training sessions to assess the impact on cognitive performance and stress response [90]. Additionally, the LifeLog Recording wearable camera automatically captures images of daily activities, helping monitor behavioral changes and supporting cognitive training programs to improve patients’ situational memory [91].

### Importance of the Caregiver’s Role

Due to limited health care resources and the growing demands of an aging population, many studies advocate for home-based caregiving to alleviate pressure on the health care system [217]. Informal caregivers play a crucial role in supporting patients with cognitive changes, yet they often face emotional stress, mental health challenges, and significant physical, psychological, and financial burdens [218-221]. Despite these challenges, the focus on informal caregivers remains limited, particularly for those caring for patients in the early stages of cognitive decline. Research has typically concentrated on more advanced stages of dementia, leaving fewer studies on caregivers in the earlier stages [222].

In the included studies, informal caregivers included spouses [67,92], children [21], siblings, and other family members [93,214]. A study by Lai et al [93] defined the informal caregiver as family members who provide daily care tasks without compensation, while Beentjes et al [214] described them as individuals living with or visiting the patient at least twice a week. Although definitions of informal caregivers varied, they generally referred to family members providing unpaid care. However, 3 studies mentioned informal caregivers without offering a clear definition, which could introduce ambiguity in interpreting the findings.

Different DHTs have demonstrated improvements for informal caregivers on multiple levels. For instance, the study by Lai et al [93] found that telemedicine interventions significantly reduced caregiver stress and were widely accepted by caregivers. Beentjes et al [214] used FindMyApp in their study to provide personalized app recommendations, not only lowering the technical barrier to use for caregivers with lower educational levels, but also transforming instrumental technology into a continuous learning platform for the development of caregiving competencies by sending relevant courses and guidelines weekly. The VR intervention in the study by Afifi et al [21] not only reactivated patients’ episodic memory by reconstructing shared virtual family scenes, but also transformed the technology into a medium for intergenerational emotional connection through a collaborative “patient-led, caregiver-followed” model. This collaborative narrative process significantly improved the mental health of both parties [21]. The study by Ghani et al [65], using the web platform, helped caregivers manage patients’ daily activities more effectively, such as medication and appointments. Although the web platform was expensive and less effective for patients with MCI, it proved cost-effective and beneficial for improving caregiver quality of life.

However, digital interventions may have unintended negative consequences. Lai et al [93] noted that telehealth interventions could weaken family dynamics, reducing problem-solving, communication, and emotional support. Additionally, not all studies showed significant improvements in caregiver outcomes. For instance, Ghani et al [65] found improvements in caregivers’ burden, but the difference was not statistically significant compared with the control group.

Future research may consider exploring digital technology-based care communities, enabling 3-dimensional collaboration among patients, caregivers, and medical teams through DHTs. For instance, distributed health records built on blockchain technology can facilitate secure cross-platform sharing of care logs; meanwhile, an analysis module integrated with AI computing can monitor patients’ emotional fluctuations in real time and trigger early warnings. Such a technological ecosystem not only supports daily care but also transforms challenges into opportunities for the whole family’s collective growth.

### Limitations

This scoping review systematically uncovered five specific research gaps in the application of DHTs for early cognitive changes: (1) long-term studies are scarce, as most research is constrained by short-term follow-up designs, making it difficult to capture long-term effects; (2) representation of patients with SCD is notably inadequate, with coverage far lower than that of individuals with MCI; (3) research on the integration of multiple DHTs is still in its early exploratory stages, lacking empirical validation; (4) personalized adaptation designs often overlook individual difference parameters such as disease progression rate, comorbidities, and user preferences; and (5) despite involving sensitive biological data, ethical and privacy risks associated with AI and IoT applications largely remain at the theoretical discussion stage, lacking empirical balancing solutions. The identification of these gaps clearly pointed out a crucial direction for future research to transition from a “technology-driven” approach to a “patient-centered” paradigm.

### Conclusions

This scoping review makes distinct contributions to the literature by systematically mapping the landscape of DHTs in early cognitive change. This scoping review’s primary contribution is threefold. First, it synthesizes evidence to reveal the significant potential of DHTs across the care continuum—from screening and diagnosis to intervention and monitoring—while critically incorporating the often-overlooked caregiver perspective, thereby offering a more holistic understanding. Second, it moves beyond a generic evaluation to delineate the differentiated clinical value of various DHTs, highlighting their unique strengths in delivering personalized interventions and maintaining continuity of care. Finally, a central challenge undermining the practical application of DHTs is the difficulty in balancing technological sophistication with user-friendliness. Looking forward, future research should focus on generating robust long-term evidence, broadening the scope to include underrepresented groups like SCD and caregivers, and developing strategies for the integrated, ethical, and truly personalized implementation of DHTs.

## Supplementary material

10.2196/82881 Multimedia Appendix 1 Search strategies.

10.2196/82881 Checklist 1 PRISMA-ScR checklist.

10.2196/82881 Checklist 2 PRISMA-S checklist.

## Abbreviations

AD: Alzheimer disease
ADL: activity of daily living
AI: artificial intelligence
AR: augmented reality
DHT: digital health technology
IoT: internet of things
MCI: mild cognitive impairment
PRISMA-S: Preferred Reporting Items for Systematic Reviews and Meta-Analyses Statement for Reporting Literature Searches in Systematic Reviews
PRISMA-ScR: Preferred Reporting Items for Systematic Reviews and Meta-Analyses Extension for Scoping Reviews
RCT: randomized controlled trial
SCD: subjective cognitive decline
VR: virtual reality
