# Supplementary material for: Digital Health Technologies Applied in Patients With Early Cognitive Change: Scoping Review
Source: J Med Internet Res. 2025 Dec 29;27:e82881. doi: 10.2196/82881 (PMC12747421; doi:10.2196/82881)
Supplement: Checklist 2 [file jmir-v27-e82881-s003.docx]

**PRISMA-S Checklist**

| **Section/topic** | **#** | **Checklist item** | **Location(s) Reported** |
| --- | --- | --- | --- |
| **INFORMATION SOURCES AND METHODS** | | | |
| Database name | 1 | Name each individual database searched, stating the platform for each. | 1, 4, and FIGURE 1 |
| Multi-database searching | 2 | If databases were searched simultaneously on a single platform, state the name of the platform, listing all of the databases searched. | 1 and 4 |
| Study registries | 3 | List any study registries searched. | 1, 4, and FIGURE 1 |
| Online resources and browsing | 4 | Describe any online or print source purposefully searched or browsed (e.g., tables of contents, print conference proceedings, web sites), and how this was done. | 4 and FIGURE 1 |
| Citation searching | 5 | Indicate whether cited references or citing references were examined, and describe any methods used for locating cited/citing references (e.g., browsing reference lists, using a citation index, setting up email alerts for references citing included studies). | FIGURE 1 |
| Contacts | 6 | Indicate whether additional studies or data were sought by contacting authors, experts, manufacturers, or others. | 4 |
| Other methods | 7 | Describe any additional information sources or search methods used. | 4 |
| **SEARCH STRATEGIES** | | | |
| Full search strategies | 8 | Include the search strategies for each database and information source, copied and pasted exactly as run. | Appendix 1 |
| Limits and restrictions | 9 | Specify that no limits were used, or describe any limits or restrictions applied to a search (e.g., date or time period, language, study design) and provide justification for their use. | 4-5 |
| Search filters | 10 | Indicate whether published search filters were used (as originally designed or modified), and if so, cite the filter(s) used. | 4 |
| Prior work | 11 | Indicate when search strategies from other literature reviews were adapted or reused for a substantive part or all of the search, citing the previous review(s). | 4 |
| Updates | 12 | Report the methods used to update the search(es) (e.g., rerunning searches, email alerts). | 4 |
| Dates of searches | 13 | For each search strategy, provide the date when the last search occurred. | 1, 4, and FIGURE 1 |
| **PEER REVIEW** | | | |
| Peer review | 14 | Describe any search peer review process. | 5 |
| **MANAGING RECORDS** | | | |
| Total Records | 15 | Document the total number of records identified from each database and other information sources. | 1, 6, and FIGURE 1 |
| Deduplication | 16 | Describe the processes and any software used to deduplicate records from multiple database searches and other information sources. | 5-6，and FIGURE 1 |

*From:* Rethlefsen, M.L., Kirtley, S., Waffenschmidt, S. et al. PRISMA-S: an extension to the PRISMA Statement for Reporting Literature Searches in Systematic Reviews. Syst Rev 10, 39 (2021). <https://doi.org/10.1186/s13643-020-01542-z>
